# Circadian regulation of dengue virus transmission and replication: insights into vector activity and viral dynamics

**DOI:** 10.3389/fcimb.2024.1482042

**Published:** 2024-09-30

**Authors:** Milad Zandi, Fatemeh Sadat Mousavi

**Affiliations:** ^1^ Department of Microbiology, Faculty of Medicine, Guilan University of Medical Sciences, Rasht, Iran; ^2^ Department of Microbiology and Immunology, Faculty of Veterinary Medicine, University of Tehran, Tehran, Iran

**Keywords:** circadian rhythms, dengue virus, *Aedes aegypti*, viral replication, vector control

## Abstract

Dengue fever, caused by dengue virus, poses a significant global health challenge, particularly in tropical regions where *Aedes aegypti* serves as the primary vector. The circadian clock *in Aedes aegypti* governs key behavioral and physiological processes, including activity patterns, feeding behaviors, and susceptibility to dengue virus infection. This article explores the influence of circadian rhythms on the mosquito’s ability to transmit dengue virus, emphasizing how the circadian regulation of gene expression, immune responses, and lipid metabolism in the mosquito vector creates temporal windows that affect viral replication efficiency.

## Introduction

Dengue fever caused by dengue virus (DENV), remains a major public health issue, especially in tropical and subtropical regions (Paz-Bailey et al., 2024). The primary vector responsible for DENV transmission is *Aedes aegypti*, a mosquito species with well-characterized circadian rhythms that govern its behavioral and physiological functions (Gentile et al., 2013; Teles-de-Freitas et al., 2020; Shetty et al., 2022). The circadian clock is an intrinsic time-keeping mechanism that orchestrates biological processes in nearly all living organisms (Kreitzman and Foster, 2011), plays a crucial role in the regulation of vector activity, feeding behaviors, and the replication of the DENV. The circadian system in *Aedes aegypti* is composed of a network of clock genes, including Period (Per), Timeless (Tim), Clock (Clk), and Cycle (Cyc), which generate oscillatory patterns of gene expression and physiological activity over a 24-hour period. These rhythms are entrained by environmental cues, primarily light and temperature, which synchronize the mosquito’s internal clock with the external environment (Ptitsyn et al., 2011). The circadian regulation of *Aedes aegypti’s* activity is critical for its role as a vector of DENV. Research indicates that *Aedes aegypti* exhibits peak activity during crepuscular periods—early morning and late afternoon—when ambient temperatures and light levels are optimal for host-seeking behavior (Day, 2016). These periods coincide with times when humans are often outdoors, thereby increasing the likelihood of vector-host contact and subsequent viral transmission (Day, 2016; Benoit and Vinauger, 2022). The circadian clock controls the expression of genes associated with locomotor activity, sensory processing, and feeding behaviors in *Aedes aegypti* (Lima-Camara et al., 2011). The expression of olfactory receptor genes, which are essential for detecting host-derived cues such as carbon dioxide and body odors, is under circadian control, leading to heightened olfactory sensitivity during peak activity periods (Shetty et al., 2024). This temporal regulation of sensory inputs ensures that the mosquito’s feeding activity is optimally timed to maximize the probability of blood meal acquisition, which is necessary for egg production and viral transmission.

## Circadian modulation of susceptibility to dengue virus

The susceptibility of *Aedes aegypti* to DENV infection is also influenced by circadian rhythms (Lima-Camara et al., 2011). Experimental studies have demonstrated that the time of day when mosquitoes are exposed to DENV can affect the efficiency of viral entry, replication, and dissemination within the vector (Alto et al., 2008). This is likely due to circadian modulation of the mosquito’s immune responses, including the production of antimicrobial peptides, reactive oxygen species, and other antiviral factors. The expression of the gene encoding the antimicrobial peptide Defensin A, which exhibits antiviral activity against DENV, oscillates in a circadian manner, potentially leading to diurnal variations in vector competence (van Spyk, 2017). Moreover, the circadian regulation of the Toll and IMD signaling pathways, which are key components of the mosquito’s innate immune response, may influence the outcome of viral infection depending on the time of exposure (Khanal et al., 2022). These findings suggest that Aedes aegypti might be more or less susceptible to DENV infection at different times of the day, which could have significant implications for the transmission dynamics of the virus.

## Circadian regulation of dengue virus replication

DENV is an RNA virus of the *Flaviviridae* family, and its replication cycle is intricately linked to the host’s cellular machinery, which itself is subject to circadian regulation (Schulze). The replication of DENV within both Aedes aegypti and human hosts is influenced by the circadian clock, which controls various aspects of cellular metabolism, immune responses, and the expression of viral host factors (Borrmann et al., 2021). The replication of DENV in *Aedes aegypti* begins with the virus entering the midgut epithelial cells after a blood meal. The virus then replicates and disseminates to secondary tissues, including the salivary glands, from where it can be transmitted to a new host (Taracena et al., 2018). Circadian rhythms in the mosquito influence this process at multiple levels. First, the circadian clock regulates the expression of genes involved in the replication of viral RNA. Components of the mosquito’s translational machinery, such as ribosomal proteins and elongation factors, exhibit circadian patterns of expression that could impact the efficiency of viral protein synthesis (Zhang and Emery, 2012). Additionally, the circadian modulation of autophagy that can either facilitate or restrict viral replication. Moreover, the temporal regulation of lipid metabolism by the circadian clock in mosquitoes could influence the availability of lipid membranes required for the assembly of the viral replication complex (Zhang and Emery, 2012; Ajayi et al., 2023). Lipid metabolism plays a critical role in the life cycle of flaviviruses like DENV, as these viruses are heavily dependent on the host’s lipid resources. The circadian clock governs not only lipid biosynthesis but also lipid storage and mobilization, impacting the supply of these essential components during different times of the day. DENV, like other flaviviruses, relies on host-derived lipid membranes to form replication organelles where viral RNA synthesis occurs. The dynamic regulation of lipid metabolism may alter the integrity and composition of these membranes, further affecting viral replication efficiency. The circadian control of lipid biosynthesis and trafficking pathways in *Aedes aegypti* may thus create temporal windows of enhanced or reduced viral replication capacity (Johnson, 2018; Borrmann et al., 2021).

## Viral replication in human hosts

Once transmitted to a human host, DENV targets various cell types, including dendritic cells, hepatocytes, and endothelial cells (Uno and Ross, 2018). The circadian regulation of host immune responses plays a significant role in shaping the outcome of infection. Interferon-stimulated genes (ISGs), which are critical for the host’s antiviral defense, are subject to circadian regulation. Studies have shown that the expression of ISGs, such as MxA and OAS1, fluctuates in a circadian manner, potentially leading to diurnal variations in antiviral activity (Los et al., 2022). This circadian regulation could influence the replication efficiency of DENV within human cells, with implications for viral load, disease severity, and the timing of peak viremia. Additionally, the circadian clock modulates the secretion of pro-inflammatory cytokines, such as IL-6 and TNF-α, which play key roles in the pathogenesis of dengue. The circadian timing of cytokine release may affect the extent of vascular leakage, a hallmark of severe dengue, thereby influencing the clinical course of the disease (Moreno-Altamirano et al., 2022; Saqallah et al., 2022). Recent studies have also identified that circadian components, including BMAL1 and REV-ERBα, influence several stages of the viral life cycle in other flaviviruses, such as hepatitis C virus (HCV), dengue, and Zika. The knockout of BMAL1 or activation of REV-ERBα inhibits viral replication via disruptions in lipid signaling pathways, which highlights the importance of lipid metabolism in DENV replication (Keating et al., 2013; Zhuang et al., 2019).

## Implications for dengue control and treatment

The optimization of vector control measures could be significantly enhanced by aligning interventions with the circadian rhythms that govern *Aedes aegypti* activity and DENV susceptibility. By synchronizing the deployment of insecticides or the release of genetically modified mosquitoes with peak periods of vector activity or heightened viral susceptibility, mosquito populations and viral transmission could be more effectively disrupted (Lees et al., 2021). In human hosts, chronotherapy could be employed, with antiviral and immunomodulatory treatments timed to coincide with circadian peaks in immune response, potentially improving therapeutic outcomes and reducing disease severity. Furthermore, leveraging Wolbachia-based interventions, which have been shown to reduce the transmission of flaviviruses like DENV, may offer additional strategies for disrupting viral replication and transmission cycles (Albertson et al., 2013; Morioka et al., 2018). Additionally, integrating circadian biology into predictive models of dengue transmission could refine outbreak forecasting, enabling more precise and timely public health interventions, which could ultimately reduce disease incidence and severity.

## Conclusion

Circadian rhythms utilize a profound influence on the behavior of *Aedes aegypti*, the primary vector of DENV, and on the replication of the virus within both the mosquito vector and human host. The temporal regulation of these processes by the circadian clock has significant implications for the transmission dynamics of DENV and for the development of effective control and treatment strategies. As research in this area continues to advance, the integration of circadian biology into dengue prevention and management programs holds the promise of reducing the global burden of this disease.

## Data Availability

The original contributions presented in the study are included in the article/supplementary material. Further inquiries can be directed to the corresponding author.
